# Crossed Renal Ectopia with a Fused Supernumerary Kidney

**DOI:** 10.7759/cureus.7669

**Published:** 2020-04-14

**Authors:** Tayeb A Rahim, Pardeep Mittal

**Affiliations:** 1 Radiology, Medical College of Georgia, Augusta University Medical Center, Augusta, USA; 2 Radiology and Imaging, Medical College of Georgia, Augusta University Medical Center, Augusta, USA

**Keywords:** supernumerary kidney, crossed fused renal ectopia, renal ectopia

## Abstract

Crossed fused renal ectopia and the presence of a supernumerary kidney are both rare congenital variants that are often asymptomatic but may be associated with other developmental anomalies. Here we present a case of a 20-year-old male with a known diagnosis of crossed fused renal ectopia as well as a history of imperforate anus and tethered spinal cord treated in infancy. He presented to the emergency room with symptoms of flank pain, and a noncontrast computed tomography (CT) scan revealed a 4-mm stone in the distal left ureter. CT scan also revealed that the patient's right kidney was not crossed and fused to the left kidney as previously believed, but rather it was crossed and fused to a supernumerary kidney abutting the inferomedial aspect of an orthotopic left kidney. This is a unique example of two rare coexisting renal anomalies not previously detected on a nuclear medicine renal scan and serial renal ultrasounds obtained earlier in the the patient's life.

## Introduction

Crossed fused renal ectopia is a rare congenital anomaly in which the kidneys are fused and are located on the same side relative to midline. The first case was reported by Dominicus Panarolus in 1654, and today, it has an estimated prevalence of about 1 in 1000 live births with a 3:2 male to female predominance [1-2].

A finding that is even less common than crossed fused renal ectopia is the presence of a supernumerary kidney, which is a distinct mass of renal parenchyma that is either completely separate from or loosely attached to a major kidney and has its own collecting system and blood supply. It was first described in 1656, and today, it has an estimated prevalence of 1 in 26750 with computed tomography (CT) imaging, without any gender predilection [3-5]. No cases of coexisting crossed fused ectopia and supernumerary kidney have been documented in the literature until now.

## Case presentation

**Figure 1:**
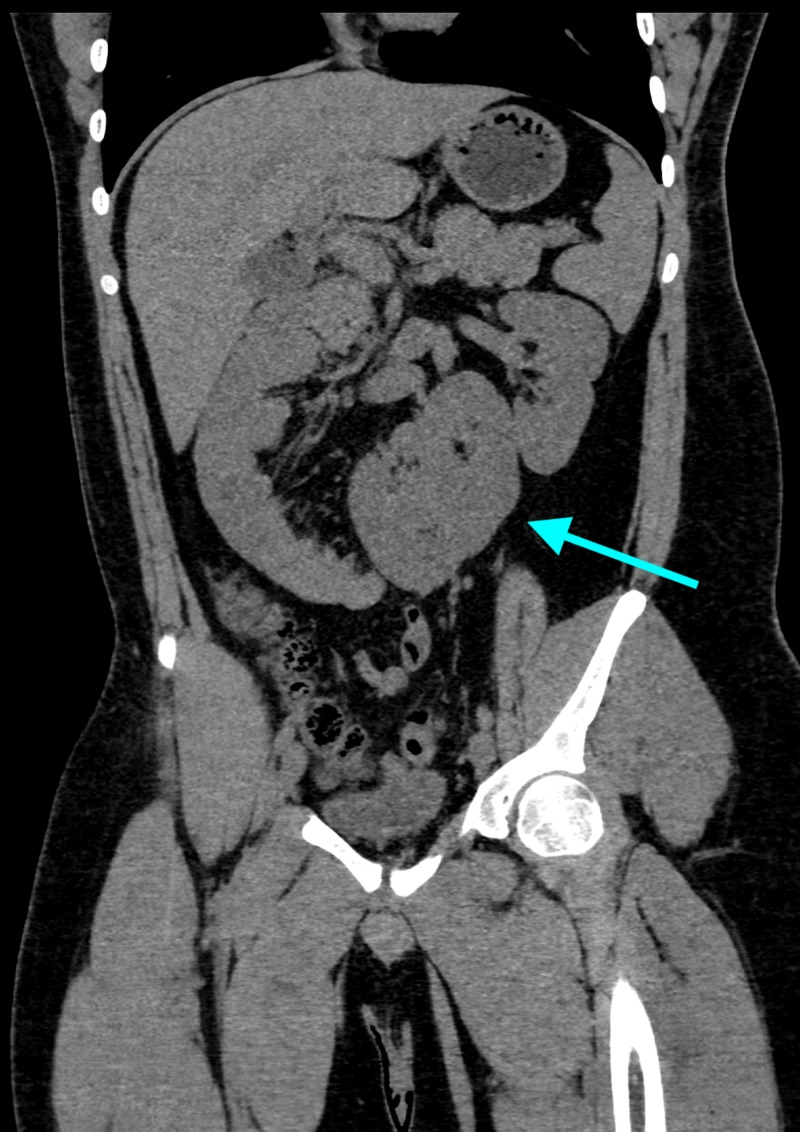
Oblique coronal image from noncontrast CT obtained on the day of presentation demonstrating a normal orthotopic left kidney and a fused ectopic moiety (arrow) abutting the inferomedial aspect of the left kidney consisting of the right kidney and a fused supernumerary kidney CT, computed tomography

A 20-year-old male presented to the emergency department with symptoms of severe left flank pain and hematuria. Non-contrast CT scan was performed, which showed a 4-mm stone in the distal left ureter. Furthermore, he was found to have crossed right renal ectopia with a fused supernumerary kidney (Figure 1, Video 1).

**Video 1:** Axial noncontrast CT through the abdomen and pelvis demonstrating a normal orthotopic left kidney and the fused ectopic moiety abutting the inferomedial aspect of the left kidney The fused ectopic moiety has two distinct renal hila, each supplied by its own artery and vein. The right renal vein arises from the inferior vena cava and the vein to the supernumerary kidney arises from the left renal vein. A 4 mm renal stone is visualized within the distal left ureter, just proximal to the ureterovesical junction, with no evidence of hydroureteronephrosis. CT, computed tomography

**Figure 2:**
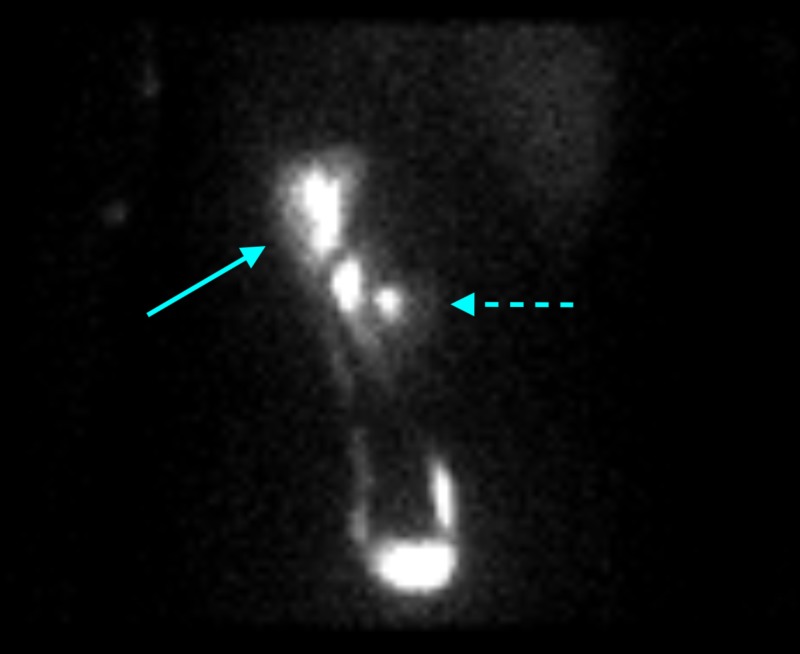
Nuclear medicine MAG3 renal scan performed about one year prior to patient's emergency department visit demonstrating an orthotopic left kidney (solid arrow) and a right renal moiety (dotted arrow) mistakenly thought to be fused to the lower pole of the left kidney Single ureters arise from each renal moiety with normal attachment to the urinary bladder. Symmetric function of both moieties was noted.

**Figure 3:**
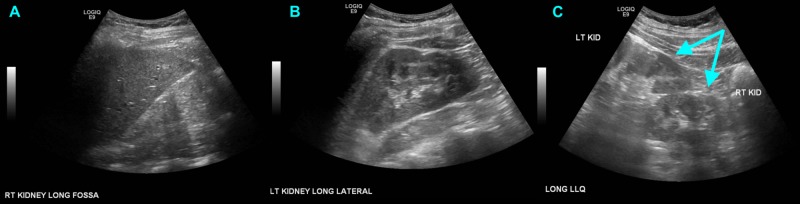
Renal ultrasound performed about one year prior to presentation demonstrating an empty right renal fossa (A), a normal appearing orthotopic left kidney (B), and a fused renal moiety (double arrow) visualized in the left lower quadrant of the abdomen, mistakenly mislabeled as "LT KID" and "RT KID" at the time of scanning (C)

It was already known that the patient had crossed fused renal ectopia based on his documented history and prior imaging examinations, However, the presence of a fused supernumerary kidney was only discovered after the CT scan was obtained. A nuclear medicine scan was performed about one year prior to patient's emergency department visit in order to assess the contribution of each kidney to the glomerular filtration rate (GFR). This showed an orthotopic left kidney and a right renal moiety mistakenly thought to be fused to the lower pole of the left kidney (Figure 2). The renal function was symmetric. In addition, the patient had several renal ultrasounds over the course of his childhood, all of which suggested the same findings. Images from his most recent renal ultrasound performed about three months prior to the aforementioned nuclear medicine are shown in Figure 3. As seen in the CT scan above, there were in fact two renal moieties: a normal left kidney and a fused right renal moiety abutting the inferomedial aspect of the left kidney, consisting of the right kidney and a supernumerary kidney.

The patient also had a history of an imperforate anus at birth, requiring a diverting descending colostomy. A year later, the patient had a Pena procedure with posterior sagittal anorectoplasty and multiple anal dilations. This followed with colostomy closure several months later. Additionally, the patient had an incidental finding on an MRI scan of spina bifida occulta with filum lipoma, for which he received an L3 laminectomy with the release of a tethered cord.

## Discussion

This case demonstrates a unique example of crossed fused renal ectopia and supernumerary kidney occurring together. Ultrasound examinations early in the patient’s life detected the presence of a fused ectopic renal moiety, but it was incorrectly thought to represent a crossed ectopic right kidney fused to the left kidney. This may be because the true diagnosis was not considered in the differential diagnosis due to its rarity.

A supernumerary kidney is thought to form when there is an abnormal division of the nephrogenic cord into two metanephric blastemas [5]. A supernumerary kidney can be distinguished from a duplex kidney by the presence of its own arterial and venous supply, pelvicalyceal system, and renal capsule. Supernumerary kidneys are more often located on the left than on the right side and may be partially fused [6-8]. Rare cases of supernumerary kidneys in conjunction with a horseshoe anomaly have also been reported [9-11]. Although the supernumerary kidney presented in this case report is fused to a crossed ectopic right kidney and drains into the right ureter, its venous drainage is into the left renal vein, suggesting a left-sided developmental origin. Aberrant leftward migration of the right ureteric bud likely led to interaction with the metanephric blastema of the supernumerary kidney to form a crossed ectopic right kidney fused to the supernumerary kidney.

Numerous associations have been made between supernumerary kidneys and other anomalies that fall into the VACTERL pattern of birth defects, an acronym which identifies a non-random co-occurrence of vertebral defects (V), anal atresia (A), cardiac defects (C), tracheoesophageal fistula (TE), renal anomalies (R), and limb abnormalities (L). These include ectopic ureteral opening, urethral or penile duplication, ureteral atresia, imperforate anus, ventricular septal defects, coarctation of the aorta, and meningomyelocele [8,12-15]. In this case, the patient had associated findings of anal atresia and spina bifida occulta. In patients with VACTERL association, there is known to be a high incidence of tethered spinal cord if the patient has anal atresia and urogenital anomalies [16]. MRI performed on this patient in infancy indeed revealed a tethered spinal cord, for which he was treated with cord release.

Most isolated cases of supernumerary kidney or crossed fused ectopia are asymptomatic and discovered incidentally [17-18]. The risk of urolithiasis in patients with crossed fused ectopia is similar to that in patients with a horseshoe kidney, with a prevalence ranging between 21 and >60% [19]. Interestingly, in this case, punctate nonobstructing stones were seen in both renal moieties, and the symptomatic urinary calculus was located in the left ureter, which arises from the normal orthotopic kidney. These findings may simply be explained by dehydration, as the patient presented during the summertime and reported low fluid intake.

The patient’s presenting symptoms of flank pain and hematuria only warranted a non-contrast CT to evaluate for stones. Since the cause of the patient's symptoms was identified with the initial non-contrast scan, the patient decided not to have a second contrast-enhanced study. Lack of intravenous contrast, unfortunately, limits full evaluation of the renal vasculature and collecting systems. However, the images that were acquired were sufficient to elucidate the patient’s gross anatomic anomalies. 

## Conclusions

Crossed fused renal ectopia and supernumerary kidneys are both rare congenital anomalies. In this case, our patient was initially diagnosed with isolated crossed fused renal ectopia, based on prior serial ultrasounds and a nuclear medicine renal scan. CT scan on the day of presentation, however, revealed a unique example of cross renal ectopia with fusion to a supernumerary kidney.
